# Leveraging AI for infectious disease modelling and public health decision making

**DOI:** 10.1186/s12919-026-00398-w

**Published:** 2026-09-21

**Authors:** Moritz U. G. Kraemer, Barbara Tornimbene, Samir Bhatt, Serina Chang, Gautam Prasad, Milind Tambe, Houriiyah Tegally, Oliver Morgan

**Affiliations:** 1 Department of Biology, University of Oxford, Oxford, UK; 2 WHO Pandemic and Epidemic Intelligence Hub, Berlin, Germany; 3 Section of Epidemiology, Department of Public Health, University of Copenhagen, Copenhagen, Denmark; 4 MRC Centre for Global Infectious Disease Analysis, School of Public Health, Imperial College London, London, UK; 5 Pioneer Centre for Artificial Intelligence, University of Copenhagen, Copenhagen, Denmark; 6 University of California, Berkeley, CA USA; 7 Google Research, Mountain View, CA USA; 8 Harvard University, Cambridge, MA USA; 9 Centre for Epidemic Response and Innovation (CERI), Stellenbosch University, Stellenbosch, South Africa

**Keywords:** Artificial intelligence, Infectious disease modelling, Epidemic intelligence, Genomic surveillance, Outbreak forecasting, Decision support, Public health preparedness

## Abstract

Artificial intelligence (AI) is expanding the capacity of public health systems to detect infectious disease signals, forecast outbreaks, analyse pathogen evolution, generate localised risk estimates, and support operational decisions. The thirteenth session of the WHO Pandemic and Epidemic Intelligence Innovation Forum brought together experts from academic, public health, and technology organisations to examine current applications of AI in infectious disease modelling and pandemic preparedness. Examples included genomic surveillance, hybrid epidemiological and machine-learning models, spatial foundation models, AI-enabled decision support, and agent-based simulations for resource allocation. Participants emphasised that technical performance alone is insufficient: tools must be transparent, auditable, transferable across settings, operationally usable, and responsive to local data and infrastructure constraints. Sustained interdisciplinary collaboration, human oversight, robust validation, and equitable design will be essential to embed AI safely and effectively within public health decision-making.

## Introduction

Artificial intelligence (AI) is transforming how we detect, model, and respond to infectious disease threats [1, 2]. From early outbreak signals to viral evolution forecasting, from genomic annotation to public health decision support, AI-enabled systems are rapidly expanding the frontiers of what is possible [1, 3, 4]. Yet, realising their full potential requires more than technological advancement. It calls for thoughtful and ethical integration into real-world contexts, where public health priorities, data realities, and decision-making timelines determine current feasibility [1, 3, 4].

The thirteenth session of the WHO Pandemic and Epidemic Intelligence Innovation Forum [5] convened experts from the University of Oxford, Harvard University, Google Research, the Centre for Epidemic Response and Innovation (CERI), the University of California Berkeley, and the University of Copenhagen/Imperial College London to explore how AI can strengthen pandemic and epidemic preparedness. While the technical potential of AI was clear across all contributions, the session also reflected a shared recognition: that tools must be trusted, auditable, usable, and actionable within the timeframes and constraints of public health decision-making.

## Detecting signals: AI for early warning in epidemic intelligence

Understanding and responding to infectious disease threats often begins with making sense of uncertainty, from detecting early signals in individuals to anticipating outbreak patterns at the population level. In this context, AI can contribute across four foundational areas: identifying risk, forecasting trajectories and imputing missing data, characterising pathogen evolution, and strengthening surveillance through adaptive, data-efficient approaches (such as active learning) [2, 6, 7].

At the individual level, AI is already used to detect early and ongoing symptoms through wearables devices that collect health data, often before individuals present to care, facilitating early detection and prompting behaviour changes that can prevent worsening of illness or transmission [8, 9]. The challenge is to scale these insights to support public health decision-making, particularly in data-scarce settings. Doing so requires tools that can adapt to evolving outbreaks, integrate diverse data types, and produce timely, context-aware outputs.

One example of this approach comes from one of the presenters’ works on a multi-layered system that combines real-time genomic surveillance, ecological modelling, and machine learning to support both rapid response and long-term forecasting [10–13]. Their approach highlights how machine learning and deep learning methods can capture complex, non-linear relationships between environmental and demographic change and pathogen spread, enabling more accurate, short- to medium-term predictions at local scales. Examples include predicting the importation risk of arboviruses such as dengue and modelling the ecological drivers of virus transmission, like Oropouche virus in South America [10, 13, 14]. The group integrated workflow illustrates the layered complexity of modelling pathogen dynamics: data sources such as genomes, epidemic time series, and climatic and demographic covariates feed into a diverse set of modelling approaches. A key part of their strategy involves mapping those classical analytical frameworks and identifying where AI can enhance speed, scalability, or resolution.

This approach aligns with a broader argument raised in the panel discussion. Several contributors noted that the most useful methods often do not replace traditional tools but combine them with AI in ways that build on their strengths. By integrating statistical models, mechanistic frameworks, and expert-driven inference with machine and deep learning techniques, these hybrid systems can deliver more robust, interpretable, and operationally relevant outputs [7]. In this view, AI acts not as a substitute but as a force multiplier, thus extending the power of established methods to meet the complexity and speed of today’s epidemic intelligence needs.

## Accelerating and democratising genomic intelligence for outbreak response

AI is also reshaping how we work with pathogen genomic data, not only by accelerating analysis but also by expanding evidence generation. Beyond lineage assignment or phylogenetic reconstruction, emerging tools are beginning to predict how mutations may affect viral properties such as transmissibility or immune escape [15]. By using deep learning and structural data to anticipate pathogen evolution we might be able to inform public health decisions in advance [16]. While still an emerging area, such approaches could help prioritise variants for laboratory validation, guide vaccine and therapeutic development, and support earlier interventions.

An applied example comes from of one of the participant’s research projects, where AI methods are being used across the genomic surveillance pipeline. For example, anomaly detection is applied to molecular clock data to flag sequences with unexpected divergence, which is a potential early signal of variant emergence [12]. Looking ahead, they plan to apply neural networks for predicting pathogen dispersal from phylogeographic data and are exploring the use of AI to support therapeutic target design directly from pathogen genomes.

Yet in many settings, these tools remain inaccessible to those who need them most. Without dedicated analytical teams or specialised expertise, public health actors in low- and middle-income countries often depend on external partners for data interpretation. But relying on external expertise can mean losing control over the analytic process, particularly during emergencies, when speed, autonomy, and trust are essential. The challenge is not just to develop powerful tools but to design them in ways that empower local users, preserve data sovereignty, and enable timely, context-specific decisions.

One example comes from a platform under development by a group of researchers from different institutions and partners across the global health community [2]. The tool supports genomic analysis in low-resource settings using large language models (LLMs) to guide users through workflows such as sequence identification, phylogenetic interpretation, and novelty detection via natural-language queries. A central principle of the design is to enable and facilitate, not dictate, ensuring users retain control over their data and analysis. The platform runs locally or in secure environments and is being co-developed with partners in LMICs to ensure usability and relevance. By embedding AI into accessible workflows, the system lowers technical barriers while expanding participation in genomic surveillance and response.

## Mapping risk: AI for high-resolution, localised inference

Beyond detecting signals, AI is also being used to bridge a persistent challenge in public health intelligence: how to translate coarse or incomplete data into meaningful, localised insights. Traditional surveillance data often lack the resolution or consistency needed for fine-grained action. AI-enabled geospatial modelling offers new methods to infer risk at subnational levels, even where direct measurement is limited [17].

A complementary approach introduced by one of the presenters utilises spatial foundation models to support epidemic intelligence where direct data may be limited or inconsistent. The Population Dynamics Foundation Model (PDFM) created by Google integrates aggregated signals, including search trends, mobility, weather, and air quality data, into a graph-based framework that produces high-resolution geospatial embeddings [18]. These can be used for disease forecasting, data interpolation, and super-resolution estimation, aiming to infer fine-grained health indicators, like ZIP-code-level disease burden, from coarser datasets such as national or county-level statistics [19, 20]. Recent examples include interpolation of health metrics in Malawi [19] and super-resolution for vaccination coverage in the US [20].

Other researchers have applied similar geospatial AI methods to estimate public health indicators at finer spatial resolutions [21]. By combining coarse administrative data with high-dimensional embeddings of search trends, mobility, and environmental variables, their models helped identify localised gaps in coverage and inform more targeted vaccination outreach.

## AI-powered decision support and resource allocation

These approaches demonstrate how AI can extend the utility of national datasets to support decision-making at the community level, especially in regions where direct data are incomplete or delayed. AI is also increasingly being used to directly support decisions from frontline operations to long-term planning. As public health systems face growing complexity, faster response expectations, and data limitations, there is a rising need for tools that help translate data into action. Although simulation and modelling have long played a role in epidemic response, classical approaches often struggle with the complexity of real-world systems, especially where behavioural, logistical, or structural factors are involved. AI-powered decision-support systems and simulation models are meeting this challenge by integrating real-world data, modelling individual behaviour, and enabling more adaptive, inclusive, and efficient public health responses [22, 23].

A participant presented a suite of tools developed to support real-time operational decisions during health emergencies. In partnership with the Virginia Department of Health during the COVID-19 pandemic, the team created interactive dashboards that integrated mobility data, point-of-interest metadata, and vaccine uptake to guide reopening policies [17, 22, 23] and optimise vaccine site placement [24]. These dashboards provided granular, real-time insights into community behaviour and access barriers, allowing decision-makers to tailor interventions with greater precision. They also developed models capable of estimating vaccine uptake at the ZIP code level by combining coarse administrative data with high-dimensional embeddings from search trends [25]. The researchers showed that vaccine uptake estimated from search trends was not only more spatially granular but also preceded official vaccination reports from the CDC by 1–2 weeks. These signals could enable more targeted and pre-emptive outreach in areas where direct coverage data were missing or delayed, helping to improve vaccination equity [26, 27].

To build on this work, the team is now using generative AI, like diffusion models and LLM-based agents, to simulate human behaviours (e.g., mobility trajectories, social networks, public opinions) and to model how individuals might respond to different public health policies [28, 29]. These synthetic populations enable public health teams to test interventions such as reopening timelines or school closures in silico before implementation. The team is also exploring how to scale these simulations for use in low-resource settings, supporting more participatory and equitable planning.

Other researchers are developing AI-powered multi-agent systems to simulate the delivery of public health interventions in complex environments. These agent-based models are designed to replicate the complex interactions between individuals, communities, and health systems, accounting for heterogeneity in behaviour, access, and structural barriers [30]. The models are built using reinforcement learning and optimisation algorithms to explore how interventions perform under different real-world conditions, such as varying supply levels, staff shortages, and geographic constraints. During the COVID-19 pandemic these models were used to compare the effectiveness of different testing strategies and demonstrated that frequent use of rapid antigen tests could outperform less frequent PCR testing in reducing transmission, despite lower test sensitivity [30]. The key insight was that speed and frequency of testing could have a greater population-level impact than diagnostic precision alone. The same simulation approach has also been applied to maternal health and HIV prevention in India, where models test how service delivery strategies perform under constraints such as limited staff, transportation barriers, or social stigma. By accounting for real-world system limitations and individual behaviours, these tools support more adaptive resource allocation, helping decision-makers identify feasible, high-impact strategies under uncertainty.

## Building trustworthy, transferable, and operational AI systems

As AI systems become increasingly embedded in public health workflows, a clear message emerged from the session: technical performance alone is not enough. For AI to support real-world decision-making, it must also be trusted, transferable, and usable. For example, during the Q&A, one attendee raised a concern about trust for AI-driven simulation tools. He pointed out that models based on known epidemiological methods are often easier to explain to decision-makers than AI systems, especially those that rely on machine learning. This opened a wider discussion about how transparency, simplicity, and communication influence trust. Speakers noted that combining classical models with AI components may be one way to help public health teams feel more confident in using these tools without losing control over decisions.

AI also needs to be capable of operating across geographies, communicating uncertainty, and complementing human judgement rather than replacing it. One central concern is the opacity of many models—so-called “black boxes”—which can erode confidence, particularly in time-sensitive or high-stakes situations. Contributors emphasised the need for transparency: clear documentation, meaningful uncertainty estimates, and user interfaces that enable practitioners to engage with and interpret model outputs. Trustworthiness, they noted, is not only a technical issue but also a relational and institutional one built through shared ownership, clear communication, and accountability between developers and users.

Another persistent challenge is the limited portability and usability of many AI tools. Models developed in high-income, data-rich contexts often fail to generalise to settings with different population structures, reporting systems, or data realities. Addressing this requires robust benchmarking, however defining appropriate benchmarks is difficult, especially where data are sparse, temporally inconsistent, or geographically misaligned. Contributors proposed several approaches to improve generalisability, including using real-world interventions as quasi-experiments and creating shared libraries of validation tasks and scenarios, particularly for foundation models applied to geospatial or behavioural inference.

Yet even well-benchmarked models can fall short if they are not operationally usable. High-performing systems often depend on infrastructure, bandwidth, or technical expertise that may be unavailable in lower-resource or emergency settings. In contrast, simpler, frugal models designed to run offline, update incrementally, and operate under local constraints may be more impactful in practice. Building AI for real-world use means striking a balance between sophistication and accessibility, ensuring that tools can function reliably in the very contexts where they are most needed.

## The road ahead: operationalising AI for future epidemic decision-making

Looking ahead, the session pointed toward a vision of AI not just as a set of tools, but as a dynamic and evolving layer within public health infrastructure. Realising this vision will require coordinated advances across modelling, data integration, system design, and institutional practice grounded in equity, transparency, and usability.

One promising direction is the application of generative AI. Diffusion models, image generators, and large language models (LLMs) are being explored for a wide range of tasks, including personalised health communication, behaviour simulation, and automated policy modelling. LLMs are increasingly being used as agents in large-scale simulations, enabling more complex modelling of human behaviour, social interaction, and information diffusion [28, 29, 31, 32]. These synthetic populations, or “digital twins”, allow decision-makers to test interventions, such as reopening strategies or vaccination campaigns, under different behavioural and structural assumptions before deployment in the real world.

Such capabilities are not only analytically powerful but also potentially transformative for policy relevance. AI systems may soon be able to identify individuals or communities at risk of disengaging from public health guidance and suggest tailored, accessible interventions to improve adherence. At the same time, influence-maximisation tools from social network theory could be used to combat misinformation, ensuring credible messages reach the right audiences more effectively. In these ways, AI could become not just a tool for analysing behaviour but a lever for influencing it and supporting more effective, inclusive, and trusted public health responses.

Another key opportunity is extending the reach of data-rich models to data-scarce settings. Contributors described how remote sensing, environmental data, and proxy indicators could be used to extrapolate population-level patterns in countries with limited internet access or incomplete surveillance infrastructure. When grounded in foundation models trained on globally diverse inputs, this approach may help fill critical gaps in real-time situational awareness. Looking ahead, future platforms could integrate forecasting, simulation, and policy scenario analysis into interactive tools that operate locally, enabling timely decisions even in settings without dedicated modelling teams [23, 33]. In such systems, large language models (LLMs) could serve as orchestration engines, linking datasets, modelling techniques, and decision-support logic to guide users through complex, high-stakes scenarios. These advances are closely tied to the growing role of passively collected, real-time data streams, such as mobility logs form mobiles phone or monitoring transportation logistics, search queries, social media activity, and environmental sensors, in informing epidemic intelligence. These sources offer fine-grained, timely insights into population behaviours without relying on self-reporting. Their utility lies not only in data richness but also in enabling public health actors to monitor on-the-ground changes, evaluate the effects of policies, and adapt interventions to local needs. When integrated with adaptive, transparent, and locally operable AI models, these data streams can help build truly operational systems that support responsive, inclusive, and equitable public health decision-making.

Speakers also pointed to several technical frontiers with high transformative potential. Structural modelling from genomic data, such as In Silico protein folding, offers previously unattainable insights into pathogen evolution and function. Graph neural networks, well-suited for modelling interdependent systems, could help capture the complexity of disease transmission, resource distribution, and institutional coordination. AI-driven inference could accelerate the use of individual-based and agent-based models in real time, overcoming the current bottleneck of model calibration and improving the usability of simulation in outbreak response.

Several contributors agreed that more work is needed to identify which use cases matter most. The forum discussion reinforced these challenges, with participants raising questions about validation across contexts, trust in black-box models, and operational constraints in resource-limited settings. A key insight emerged: identifying which AI applications deliver the greatest public health impact remains context dependent. One step to help move in this direction could be a cross-organisational survey to identify high-value AI use cases for infectious-disease control. This would aim to map current challenges across surveillance, clinical care, laboratory work, and policymaking, and rank areas where AI could add practical value. The design would combine structured scoring on feasibility, data readiness, risk, and cost with inputs from technical and decision-making teams across sectors. The results could support shared planning, identify pilot opportunities, and provide an evidence-based view of where AI can improve speed, coverage, and fairness in epidemic response. Ultimately, the session pointed toward a shared vision: AI as a scalable, ethical, and embedded layer within public health systems. Realising that vision will require sustained collaboration across disciplines, transparent processes for validation and use, and a commitment to equity at every stage of development and deployment. In this view, operational AI is not a final product, it is a system in motion, shaped by trust, alignment, and shared learning. Moving forward, deeper integration between public health programmes, data teams, and AI developers will be essential to ensure that tools are not only technically sound, but relevant and usable at the point of need.

## Data Availability

Not applicable.

## List of abbreviations

AI: Artificial intelligence
CDC: Centers for Disease Control and Prevention
CERI: Centre for Epidemic Response and Innovation
LLM: Large language model
LMIC: Low- and middle-income country
PCR: Polymerase chain reaction
PDFM: Population Dynamics Foundation Model
WHO: World Health Organization
ZIP: Zone Improvement Plan
